# Acute Soft Head Syndrome, A Rare Complication of Sickle Cell Anemia: A Case Report

**DOI:** 10.1002/ccr3.72665

**Published:** 2026-05-11

**Authors:** Mohammed Nuru‐Deen Fuseini, John G. G. Banin, Suabir Sulemana

**Affiliations:** ^1^ Department of Emergency Medicine AngloGold Ashanti Health Foundation Obuasi Ghana; ^2^ Department of Internal Medicine AngloGold Ashanti Health Foundation Obuasi Ghana

**Keywords:** acute soft head syndrome, hematopoiesis, intracranial involvement, sickle cell disease, skull bone infarction, subgaleal hematoma

## Abstract

Acute soft head syndrome (ASHS) is a rare clinical complication of sickle cell disease (SCD). It presents as a progressive, non‐traumatic, and tender swelling of one or more areas of the scalp. Aetiopathophysiology is still less understood but hypothesized to be a result of loss of cortical skull bone integrity from increased extramedullary hematopoiesis. Diagnosis is clinically made. However, imaging is very necessary to rule out an intracranial involvement, commonly an epidural hematoma. ASHS with no intracranial involvement is managed conservatively. We present the case of a 15‐year‐old adolescent living with SCD who presented with non‐traumatic tender swellings of the scalp with no other form of SCD crises. A non‐contrast head CT scan was done, which reported blood collection in the subgaleal space of the frontal and occipital regions with no associated bone fractures and intracranial bleeds. Both the patient and his parents were counseled on the condition and the need for admission. The patient received empirical antibiotics, adequate analgesia, hydration, and adequate bed rest for a total of 6 days on admission. There was marked resolution of the swelling on the day of discharge. ASHS is a rare clinical entity with limited literature and awareness. The aim of this case report is to impact knowledge and create awareness of ASHS.

## Key Clinical Message

Acute soft head syndrome (ASHS) is a rare complication of sickle cell disease presenting as tender scalp swelling. Diagnosis is clinical, supported by imaging to exclude intracranial bleeds. Conservative management is effective in cases without intracranial involvement. This case highlights ASHS's recognition and successful non‐surgical treatment in a teenage patient and underscores the value of educating healthcare providers to improve patient outcomes in this uncommon condition.

## 1 Introduction

Sickle cell disease (SCD) is a genetically inherited hemoglobinopathy. It is inherited in an autosomal recessive Mendelian trait. Meaning, both parents need to at least be carriers to possibly have an offspring with SCD. At the molecular level, the pathophysiology of the disorder stems from the substitution of glutamate for valine codon at the sixth position of the genetic code for beta‐hemoglobin [1]. The result of this mutation is an insoluble and unstable beta‐hemoglobin that cannot withstand stress or precipitants of SCD crises like dehydration, hypoxia, infection, and acidosis. SCD patients suffer a myriad of complications, including hemolytic crisis, vaso‐occlusive pain episode (VOPE), sequestration crisis, aplastic crisis, and avascular necrosis of the head of the femur. Most of these common complications have received detailed discussions in the literature on SCD owing to their high prevalence [2, 3]. Nonetheless, acute soft head syndrome (ASHS) is a very rare complication of SCD with limited awareness and knowledge on its pathophysiology, clinical features, and management [4].

ASHS, also known as acute soft skull syndrome, presents as an acute non‐traumatic tender swelling in one or more regions of the head/scalp in patients with SCD [5]. It may occur in isolation or concurrently with other forms of crises. The pathophysiology of ASHS is akin to that of avascular necrosis of the head of the femur because both stem from microvascular occlusions leading to bony infarctions. In the case of ASHS, it is believed to result from cortical skull bone infarction leading to pooling of blood and marrow content from the skull bone marrow into the subgaleal space [6, 7]. As of 2021, the World Health Organization (WHO) estimated a global prevalence of SCD at 7.74 million with 80% from sub‐Saharan Africa [8]. In Ghana, SCD is becoming a public health problem as about 15,000 Ghanaian newborns representing 2% are diagnosed annually. Out of this number, 55% are having the homozygous form [9]. Although the prevalence of SCD is relatively increasing, there is still limited awareness of ASHS as a complication of SCD among health professionals, likely because of the paucity of case reports from various parts of the globe. To the best of our knowledge, this is the second documented case in Ghana following a similar case reported by Emmanuel et al. [10] at 37 Military Teaching Hospital. This case report provides us an opportunity to contribute to the limited literature on ASHS and at the same time, create awareness and impact knowledge on its clinical presentation, diagnosis and management.

## 2 Case Presentation

### 2.1 History and Physical Examination

We present the case of a 15‐year‐old male junior high school graduate living with SCD, genotype HbSS. He is a regular attendant of the sickle cell clinic at the AngloGold Ashanti Health Foundation (AGAHF), Obuasi in the Ashanti region of Ghana. The patient is compliant on his routine medications, including hydroxyurea 750 mg daily, which was prescribed to him one and a half month prior to presentation, folic acid 5 mg daily, zincovit, and penicillin V. Per the hospital's records, he has been admitted four times within the last 4 years for various forms of crises, including acute chest syndrome, acute osteomyelitis, and VOPE. This index presentation to the emergency unit was quite different from previous presentations and was also strange to health professionals because of its rare nature. He presented with a 2‐day history of progressively tender multiple swellings on his head associated with generalized headache (Pain Severity Score of 5/10). Direct questions were negative for recent trauma, family or personal history of bleeding disorder, and any form of abuse. Recorded vitals on presentation, blood pressure: 126/81 mmHg, pulse: 108 bpm, temperature: 36.5°C, respiratory rate of 23 cpm, and saturating at 98% on room air.

**FIGURE 1:**
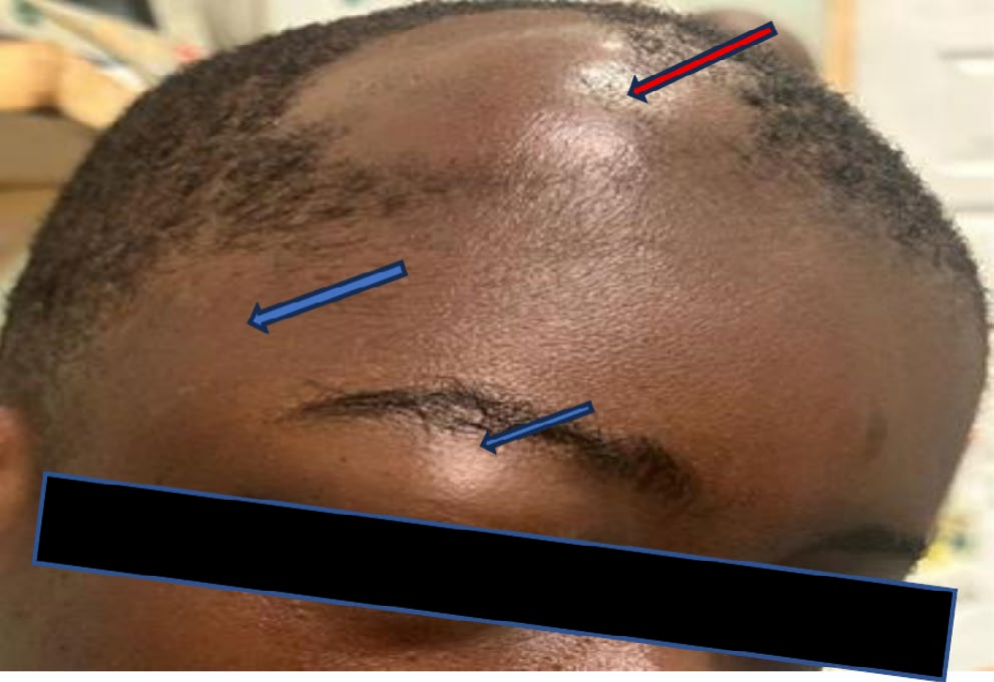
Photograph of patient showing swelling scalp at the frontal and temporal regions and upper eyelids.

On general examination, the patient appeared acutely ill and anxious, not pale, not jaundiced, and was not warm to the touch. Systemic examinations were essentially normal. On head and neck exam, there were multiple tender swellings on the left occiput and right frontal regions with extensions to the right temporal and upper eyelids. The right frontal, temporal, and upper eyelid swellings were tender, boggy, and communicating with irregular margins, consistent with subgaleal hematoma (SGH) (Figure 1). On deep palpation, there was a rim‐shaped depression measuring 3 cm x 2.5 cm on the right frontal skull bone. However, the occipital swelling was well‐organized, mildly tender, firm, non‐fluctuant, and had well‐defined margins, measuring 5 cm × 7 cm. There were no palpable lymph nodes. Considering the history and physical examination, ASHS was suspected.

### 2.2 Clinical Investigations

ASHS can be diagnosed clinically with a high index of suspicion in patients living with SCD. However, blood tests and images can help confirm the diagnosis of ASHS and to rule out other conditions that may seemingly present like ASHS. Full blood count (FBC), Liver function test (LFT), Renal function test (RFT), urinalysis, skull X‐ray, and non‐contrast enhanced CT of the head were carried out for the patient. FBC revealed hemoglobin level of 8.7 g/dL (11.0–18.0 g/dL), white blood cell also counts of 17.52 × 10^9^/L (2.5–8.5 × 10^9^/L) with neutrophilia (13.19 × 10^9^/L) and platelet count of 372 × 10^9^/L (150–400 × 10^9^/L). Creatinine was 56.3 μmol/L (60–125 μmol/L) and urea of 2.4 mmol/L (1.7–8.3 mmol/L). All the parameters for both LFT and urinalysis were also within normal limits.

**FIGURE 2:**
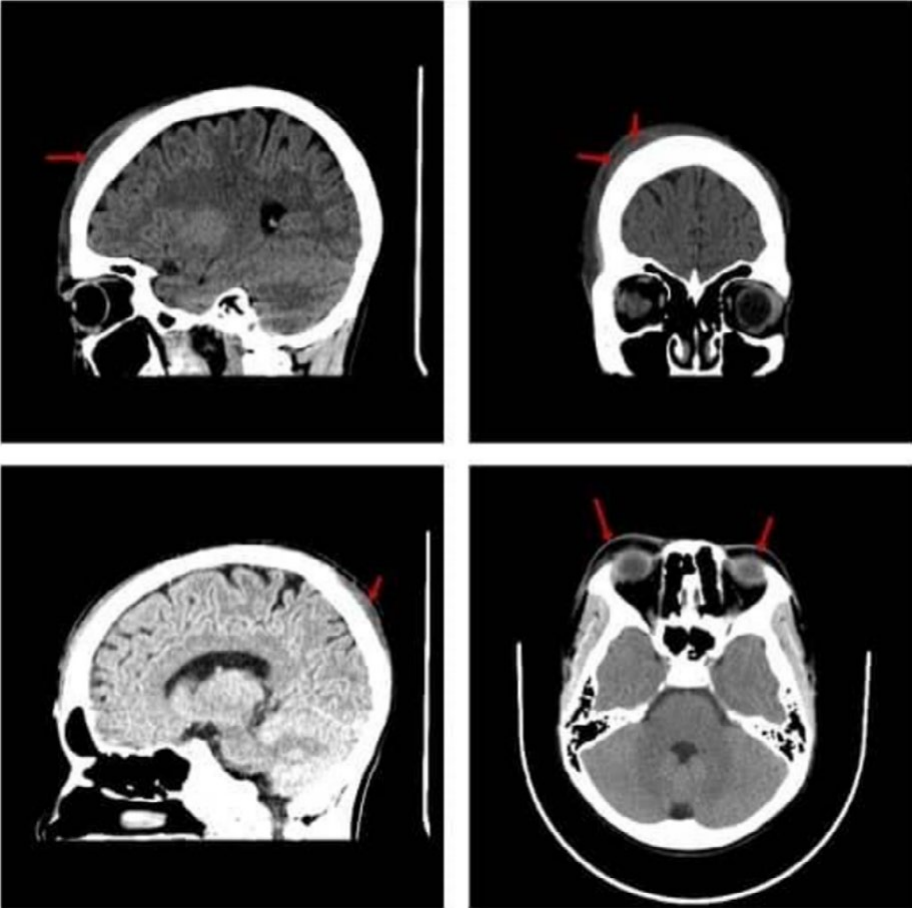
Non‐contrast enhanced head CT scan showing subgaleal hematoma (red arrows) of the right frontal and occipital scalp regions, extensions to the right temporal and bilateral upper eyelids.

Skull X‐ray was unrevealing. However, a non‐contrast‐enhanced CT scan of the head reported an encapsulated subcutaneous scalp collection in the right frontal and left occipital, likely SGH with extensions to the right temporal and periorbital. There was no evidence of intracranial hemorrhages (ICH) and skull fractures (Figure 2).

### 2.3 Diagnosis and Management

The differential diagnoses of swollen scalp may include abscess, trauma, non‐accidental injuries, tumors, and ASHS. The patient lived with the parents and did not express any concern about abuse either from them or a third party. He did not report any recent trauma as well. There is no family history of bleeding disorders. Given the features of the swelling (tender, non‐fluctuant with no differential warmth), an abscess was less likely. Also, given the sudden onset of the swelling, a tumor was also unlikely. In view of that, the diagnosis of ASHS was made.

Following the diagnosis of ASHS, both the patient and parents were counseled and reassured on the treatment modality on the basis of the tentative guidelines of the available literature on ASHS. He was admitted to the males' medical ward when the parents consented. The patient received empirical antibiotics (IV ceftriaxone 2 g daily, IV clindamycin 300 mg six‐hourly, and IV paracetamol 1 g eight‐hourly), adequate analgesia, hydration, and strict bed rest.

**FIGURE 3:**
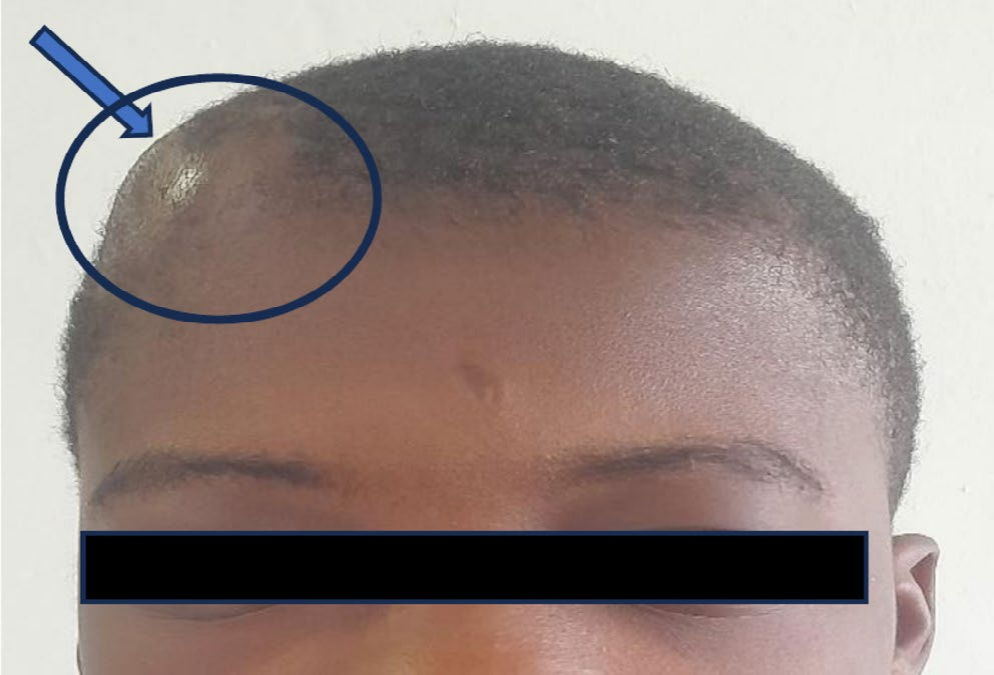
Photograph of a patient showing a well‐organized swelling of the right frontal scalp.

He spent a total of 6 days on admission with daily reviews and monitoring of the sizes of the swellings. On the day of discharge, swellings were observed to have resolved markedly except the frontal scalp, which had not completely resolved, and this time it was firm and non‐tender (Figure 3). His routine medications were refilled, antibiotics were converted to orals, and he was given a short review date of a week at the physician specialist's clinic.

### 2.4 Follow‐Up

**FIGURE 4:**
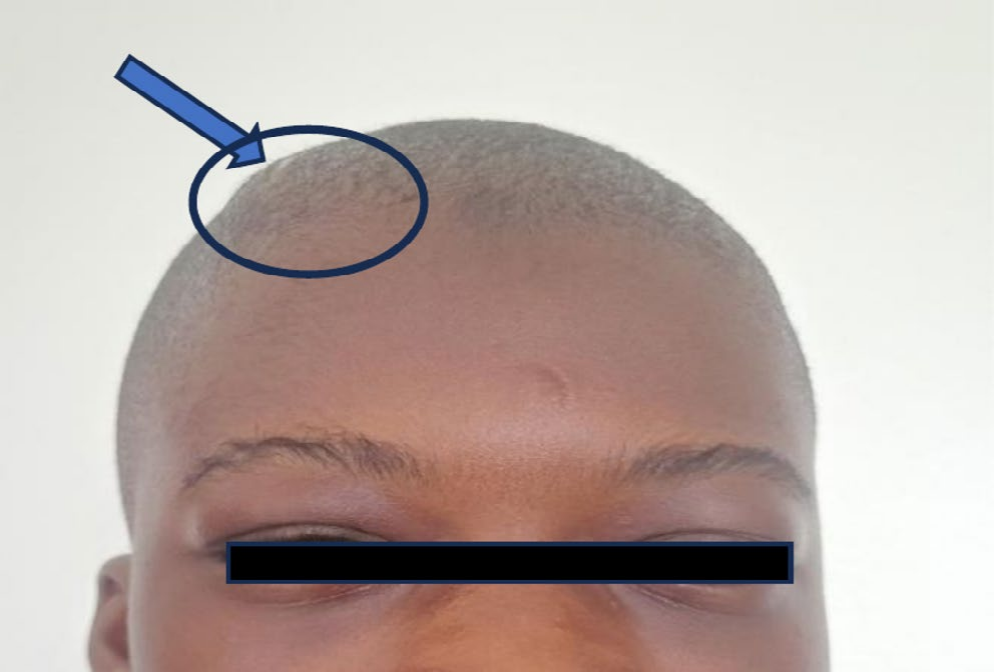
Photograph of a patient showing a small swelling of the right frontal scalp.

One week after discharge, the patient presented to the clinic for review without new complaints. General and systemic examination was essentially normal. On head examination, there was complete resolution of the swelling except for a firm, non‐tender little bump on the frontal scalp (Figure 4). He was scheduled for review the next week and to continue his routine medications.

On the second week, he presented for review at the clinic without new symptoms. On assessment, the patient had normal vitals and examination with total resolution of scalp swellings. Subsequent reviews were unremarkable. On the monthly review, he was also assessed for complications of hydroxyurea through clinical and laboratory assessments. Both history and physical examination were unrevealing. Also, FBC, LFT, and RFT panels were within normal range.

## 3 Discussion

SCD is an inherited autosomal recessive hemoglobinopathy. Sickle cell disease, as the name suggests, is characterized by abnormal deformation of a normal biconcave red blood cell (RBC) into an abnormal sickle‐shaped RBC in the setting of hypoxia from physiological stress. These deformed RBCs undergo hemolysis and polymerize into clots that block blood vessels, resulting in the various SCD complications [11].

The complications of SCD are numerous and vary in severity from patient to patient. Common complications of SCD include hemolytic crisis, VOPE, sequestration crisis, and avascular necrosis of the head of the femur from infarction. Despite the well‐documented literature on SCD complications, ASHS still remains a rare and misdiagnosed complication of SCD [12]. Since being diagnosed with SCD, our patient has experienced only acute chest syndrome, acute osteomyelitis, and multiple VOPE. However, this was his first time exhibiting features of ASHS, which increases the likelihood of misdiagnosis. ASHS presents as an acute, non‐traumatic tender scalp swelling resulting from extravasation of blood into the subgaleal space [12]. Although the aetiopathological explanation of ASHS is not fully understood, there are various postulations that report that it is a result of skull bone cortical thinning and disruption from infarctions. It is believed that increased intramedullary hematopoietic tissue disrupts both the inner and outer cortices of the skull [13]. This disruption affects the local blood supply to the skull bones, resulting in bone infarction and necrosis [14]. Our index case was aged 15 years, which is within the 11–20 years age range, which is common for ASHS. This is a period of growth spurt and hormonal surge, likely testosterone [15]. It has been proposed that the rise in testosterone during adolescence generates stress via bone remodeling and hematopoiesis, compromising cortical skull integrity and predisposing to infarction [16]. Consequently, an abnormal communication is created between the skull intramedullary space and subgaleal space or the epidural space, leading to extravasation of blood and marrow content into these spaces [17]. The abnormal communication in our case was most likely the rim‐shaped, possibly an infarcted part of the frontal scalp through which the blood flowed from the intramedullary space into the subgaleal space. ASHS can present in isolation or can co‐occur with an intracranial involvement or bleed, most commonly an epidural hematoma if the inner cortex of the skull bone is also disrupted. Our case reported only ASHS, which is consistent with many case reports; however, Drew et al. [18] reported both ASHS and EDH in the same patient.

The diagnosis of ASHS can be made clinically [12]. Garba et al. [19] reported a case where diagnosis of ASHS was made clinically and managed conservatively with an excellent outcome without imaging on the basis of financial constraints. Albeit, it is imperative to undertake imaging studies to detect co‐existing intracranial involvement, which may inform the mode of treatment [12]. Although both CT and MRI scans have places as diagnostic tools for ASHS, MRI is widely preferred for its high sensitivity [20]. However, because of cost and availability, most would resort to the use of a CT scan.

In our index case, we had no MRI in our facility, coupled with concerns of financial constraints from the parents, so we opted for a CT scan, which was readily available. In situations where any imaging cannot be afforded, like in the case of Garba et al. [19], patients should be examined thoroughly for neurological deficits and increased intracranial pressure (ICP). The presence of neurological deficits and/or increased ICP can serve as surrogates for identifying intracranial involvement.

In the absence of a co‐existing intracranial involvement, the management of ASHS is solely conservative. However, in rare instances, surgical interventions may be required because of intracranial involvement [21]. In this case, imaging did not reveal intracranial involvement, so conservative management was achieved through empirical antibiotics for superimposed infection and because of the high risk of infection in the SCD state, adequate hydration, strict bed rest, and intermittent inspection of the swellings.

Patients who experience an episode of ASHS have a higher risk of another ASHS. Frequent and long‐term follow‐up is prudent. In our index case, the patient was scheduled to be reviewed weekly for a month and then monthly thereafter.

## 4 Conclusion

ASHS still remains extremely rare and a potentially misdiagnosed clinical complication of SCD, with limited data on its global prevalence. In this context, it should be incorporated into the scope of health professional education to enhance awareness in the future. With a conservative approach, swellings can resolve spontaneously and completely with excellent outcomes. However, primary healthcare settings with no imaging modalities should refer to higher centers where intracranial involvement can be ruled out before deciding on management. Knowledge and awareness of ASHS are still limited among healthcare providers and patients; therefore, this case report aims to educate and heighten awareness of the condition, which goes a long way toward enhancing prompt diagnosis, timely intervention, and improved patient outcomes.

## Author Contributions

**Mohammed Nuru‐Deen Fuseini:** conceptualization, writing – original draft, writing – review and editing. **John G. G. Banin:** project administration, writing – review and editing. **Suabir Sulemana:** writing – review and editing.

## Funding

The authors have nothing to report.

## Ethics Statement

Ethics approval was obtained from hospital management and the ethics committee.

## Consent

Written informed consent was obtained from the patient's parents for publication of this report.

## Conflicts of Interest

The authors declare no conflicts of interest.

## Data Availability

The raw data pertaining to this case report are available on reasonable request.

## References

[1] R. D. Micheal , J. F. Melissa , and P. V. Elililoff , “Heamoglobinopathies,” in Nelson Textbook of Paediatrics, 20th ed., ed. K. S. Robert and F. S. Bonita (Elsevier, 2015), 1663–1665.

[2] M. R. DeBaun , L. C. Jordan , A. A. King , et al., “American Society of Hematology 2020 Guidelines for Sickle Cell Disease: Prevention, Diagnosis, and Treatment of Cerebrovascular Disease in Children and Adults,” Blood Advances 4, no. 8 (2020): 1554–1588. 32298430 10.1182/bloodadvances.2019001142 PMC7189278

[3] A. M. Brandow , C. P. Carroll , S. Creary , et al., “American Society of Hematology 2020 Guidelines for Sickle Cell Disease: Management of Acute and Chronic Pain,” Blood Advances 4, no. 12 (2020): 2656–2701. 10.1182/bloodadvances.2020001851 PMC7322963 32559294

[4] R. M. Emily and L. M. Jeffery , “Sickle Cell Aneamia,” Drug 72 (2012): 895–906.

[5] N. A. Alli , R. D. Wainwright , D. Mackinnon , S. Poyiadjis , and G. Naidu , “Skull Bone Infarctive Crisis and Deep Vein Thrombosis in Homozygous Sickle Cell Disease‐Case Report and Review of the Literature,” Hematology 12 (2007): 169–174. 17454200 10.1080/10245330601111912

[6] S. O. Akodu , O. F. Njokanma , I. N. Diaku‐Akinwumi , P. O. Ubuane , and U. O. Adediji , “Acute Soft Head Syndrome in Children With Sickle Cell Aneamia in Lagos, Nigeria,” Indian J Heamatol Blood Transfus 30 (2014): 67–69. 10.1007/s12288-013-0251-6 PMC4192213 25332539

[7] R. Y. Al‐Ansari , M. Al Harbi , N. Al‐Jubair , et al., “Acute Soft Head Syndrome (Subgaleal Haema Toma) With Periorbital Oedema as a Rare Presentation in Sickle Cell Disease,” Eur J Case Rep Intern Med 7, no. 10 (2020): 001766. 33083354 10.12890/2020_001766 PMC7546570

[8] WHO , “Sickle‐Cell Disease: A Strategy for the WHO African Region,” (2021), arf/rc60/8.

[9] K. Ohene‐Frempong , J. Oduro , H. Tetteh , and F. Nkrumah , “Screening Newborns for Sickle Cell Disease in Ghana,” Pedi‐Atrics 121 (2008): S120–S121.

[10] E. P. Abbeyquaye , N. Gyapong‐Osei , T. O. Peprah , et al., “Painful Nontraumatic Head Swelling in a Child With Known Sickle Cell Disease: A Case Report,” Journal of Sickle Cell Disease 2, no. Supplement_1 (2025): yoaf013.022, 10.1093/jscdis/yoaf013.022.

[11] C. Elendu , D. C. Amaechi , C. E. Alakwe‐Ojimba , et al., “Understanding Sickle Cell Disease: Causes, Symptoms, and Treatment Options,” Medicine 102, no. 38 (2023): e35237, 10.1097/md.0000000000035237. 37746969 PMC10519513

[12] N. F. Magitta , F. B. Komanya , B. O. Alphonce , M. D. Bitesigilwe , E. M. Sindato , and J. R. Meda , “Acute Soft Head Syndrome in a Teenager With Sickle Cell Anemia: A Case Report,” Clinical Case Reports 11 (2023): e8174, 10.1002/ccr3.8174. 37942183 PMC10628107

[13] V. C. Ejindu , A. L. Hine , M. Mashayekhi , P. J. Shorvon , and R. R. Misra , “Manifestations of Musculoskeletal Disease,” Radiographics 27, no. 4 (2007): 1005–1021. 17620464 10.1148/rg.274065142

[14] S. Arends , J. A. Coebergh , J. L. Kerkhoffs , A. van Gils , and H. Koppen , “Severe Unilateral Headache Caused by Skull Bone Infarction With Epidural Haematoma in a Patient With Sickle Cell Disease,” Cephalalgia 12 (2011): 1325–1328. 10.1177/0333102411414441 21803935

[15] S. Mohammed , A. H. Foula , A. AlQurashi , A. Alsaihati , and M. Sharroufna , “Spontaneous Subgaleal Hematoma in a Patient With Sickle Cell Disease: A Case Report and Literature Review,” Clinical Case Reports 7, no. 11 (2019): 2220–2224. 31788283 10.1002/ccr3.2435 PMC6878059

[16] G. Maor , Y. Segev , and M. Phillip , “Testosterone Stimulates Insulin‐Like Growth Factor‐I and Insulin‐Like Growth Factor‐I‐Receptor Gene Expression in the Mandibular Condyle—A Model of Endochon Dral Ossification,” Endocrinology 140, no. 4 (1999): 1901–1910. 10098530 10.1210/endo.140.4.6618

[17] A. Mahmud , F. E. Ahmed , and S. Mahmud , “Sickle Cell Aneamia: Image From Head to Toe,” Egyptian Journal of Radiology and Nuclear Medicine 44 (2013): 547–561.

[18] M. Drew , D. Fladeland , and R. Sinha , “Acute Soft Head Syndrome in Sickle Cell Anemia: Creating a Firm Approach,” J Haematol Thromb Dis 11, no. 3 (2023): 535.

[19] N. A. Garba , I. Ahmadu , M. S. Abubakar , M. O. Asani , and I. Aliyu , “Acute Soft Head Syndrome in Sickle Anemia: The First Case Report in Kano,” Med J DY Patil Vidyapeeth 15 (2022): 412–414.

[20] C. Zadeh , V. Rameh , and L. A. Atweh , “Acute Soft Head Syndrome in a Sickle Cell Disease Patient,” J Radiol Case Rep 15, no. 4 (2021): 1–6. 10.3941/jrcr.v15i4.4026 PMC8253147 34276870

[21] C. E. Nelson and R. J. Scarfone , “A Teenager With Sickle Cell Disease and Scalp Swelling,” Pediatric Emergency Care 34, no. 9 (2018): e168–e170. 28953104 10.1097/PEC.0000000000001295

